# Gastrointestinal nematode control practices on lowland sheep farms in Ireland with reference to selection for anthelmintic resistance

**DOI:** 10.1186/2046-0481-64-4

**Published:** 2011-03-31

**Authors:** Thomas Patten, Barbara Good, James P Hanrahan, Grace Mulcahy, Theo de Waal

**Affiliations:** 1 UCD School of Agriculture, Food Science and Veterinary Medicine, University College Dublin, Dublin 4, Ireland; 2 Teagasc, Animal Production Research Centre, Athenry, Co Galway, Ireland

## Abstract

Gastrointestinal parasitism is a widely recognised problem in sheep production, particularly for lambs. While anthelmintics have a pivotal role in controlling the effects of parasites, there is a paucity of data on how farmers use anthelmintics. A representative sample of Irish lowland farmers were surveyed regarding their parasite control practices and risk factors that may contribute to the development of anthelmintic resistance. Questionnaires were distributed to 166 lowland Irish sheep producers. The vast majority of respondents treated their sheep with anthelmintics. Lambs were the cohort treated most frequently, the majority of farmers followed a set programme as opposed to treating at sign of disease. A substantial proportion (61%) administered four or more treatments to lambs in a 'normal' year. Departures from best practice in anthelmintic administration that would encourage the development of anthelmintic resistance were observed. In conclusion, in the light of anthelmintic resistance, there is a need for a greater awareness of the principles that underpin the sustainable use of anthelmintics and practices that preserve anthelmintic efficacy should be given a very high priority in the design of helminth control programmes on each farm. To this end, given that veterinary practitioners and agricultural advisors were considered to be the farmer's most popular information resource, the capacity of these professions to communicate information relating to best practice in parasite control should be targeted.

## Background

The contribution of helminth parasites to production and economic losses in ruminant production systems is widely recognised. Commercial sheep farming in Ireland, with the emphasis on meat production, is predominantly a lowland grassland-based system. In this context the most important parasites for grazing lambs are the gastrointestinal nematodes; primarily *Nematodirus battus, *which can result in high mortality, and *Teladorsagia circumcincta *plus *Trichostrongylus *species, which can cause substantial losses in productivity through lower weight gain. The advent of effective broad-spectrum anthelmintics to the marketplace meant that sheep producers could effectively control the negative impact that parasites have on performance. However, the development of anthelmintic resistance in parasitic populations threatens this approach. Treatment frequency, the proportion of the population exposed to the anthelmintic, inappropriate dose rate and movement of sheep containing drug resistant worm populations are considered important factors influencing the rate of development and prevalence of drug resistance [1-3].

While expenditure on anthelmintics for livestock in Ireland accounts for approximately 25% of the animal health market [4], there is no information available on the cost of specific parasite control practices for sheep. With some evidence of anthelmintic resistant parasites in Ireland [5-8] and no published information on parasite control strategies used by Irish sheep producers, the purpose of this study was to obtain information on parasite control strategies on lowland sheep farms that would inform dialogue on sustainable parasite control practices. The results of the questionnaire are outlined with emphasis on farm details and on grazing management and parasite treatment practices.

## Methods

### Questionnaire

A questionnaire relating to farm details, grazing management and parasite treatment practices was designed. Prior to administering the final draft to the target population the questionnaire was piloted to ten Teagasc (Irish Agriculture and Food Development Authority) staff members, for feedback and subsequent amendment. Questions about farm profile related to the previous year while questions on parasite control practices related to typical practice in a 'normal' year.

The questionnaire (38 questions) was divided into two sections. Section 1 incorporated general questions relating to the farm and the management system (e.g., farm size, number and breed of sheep, grazing and meal feeding practices). Section 2 contained specific questions on practices for control of gastrointestinal parasites (e.g., frequency, timing and type of treatment and factors governing the choice of anthelmintic used).

### Selection of farms

The target population in this study was sheep producers (clients of Teagasc) where sheep formed a major part of the farm enterprise for a long period and who had more than 100 breeding ewes. A total of 166 farmers were selected for this study and were either from sheep producers already involved in a Teagasc study, on technology evaluation and transfer [9] or from farmers identified by Teagasc advisors. Advisors located throughout the country were asked to nominate three to five farms from their region.

### Data management and statistical analysis

Data were entered in duplicate on Excel spreadsheets (unanswered questions were left as blank) and screened using SAS procedures [10]. Any anomalies were checked and corrected.

Results are presented as percentages. The absolute numbers on which the percentages were based are in parentheses.

## Results

### Response to questionnaire

Questionnaires were returned by 70% of recipients (n = 117 respondents). However, 13 respondents were subsequently excluded from analysis as the number of breeding ewes per flock was either less than 100 (n = 7) or information was missing (n = 6). In all cases the reported percentages represent the percentage of those who responded to the question.

### Farm profile

Livestock and enterprise details are summarised on Tables 1, 2, and 3. The majority of respondents (63% [65]) had both a sheep and cattle enterprise (Table 1). Suffolk was the dominant breed with Suffolk cross ewes and Suffolk rams in 41% and 42% of flocks, respectively (Table 2). Flock replacements were homebred on 51% (58) of farms. The majority of respondents 96% (99) housed their sheep during the winter. March was the most frequently selected month for lambing, with 83% (86) of all farms indicating it as one of their target lambing months (January 14% [15], February 27% [28], March 83% [86], April 34% [35], May 4% [4]). Set stocking was reported on 19% (19) of all farms. Rotational grazing was the most popular system, and was reported on 78% (80) of all farms; the remaining 3% (3) incorporated both systems into their grazing plan (Table 3). Where the enterprise was cattle plus sheep, the majority, 72% (46), practiced mixed grazing.

**Table 1:** Farm parameters, number of farms, means and ranges on the different types of enterprise

	Sheep only		Sheep and Cattle		All enterprises	
	
	Mean (n)	Range	Mean (n)	Range	Mean (n)	Range
**Sheep**						
LSU/ha	2.10 (39)		1.65 (65)		1.82 (104)	
Flock size (breeding ewes)	446 (39)	117-1250	342 (65)	105-2000	381 (104)	105-2000
Lambs purchased for: Finishing						
Finishing	374 (5)	40-780	276 (9)	23-500	311 (14)	23-780
breeding						
replacements	85 (20)	16-165	59 (38)	20-136	68 (58)	16-165
**Cattle**						
Number of cows	NA	NA	43 (55)	1-140	43 (55)	1-140
Number of cattle:						
< 1 year	NA	NA	43 (54)	8-150	43 (54)	8-150
1-2 years	NA	NA	40 (54)	4-200	40 (54)	4-200
>2 years	NA	NA	12 (33)	1-150	12 (33)	1-150

**Table 2:** Ewe and ram breed types on the different types of enterprise

	Sheep only (n = 39)	Sheep and Cattle (n = 65)	All enterprises (n = 104)
	% (n)	% (n)	% (n)
**Ewe breed**			
Mostly Suffolk cross	41 (16)	42 (27)	41 (43)
Mostly Texel cross	8 (3)	2 (1)	4 (4)
Other	3 (1)	9 (6)	8 (7)
Combination	49 (19)	48 (31)	48 (50)
			
**Ram breed**			
Mostly Suffolk	50 (14)	52 (30)	42 (44)
Mostly Texel	39 (11)	26 (15)	25 (26)
Both	11 (3)	22 (13)	15 (16)

**Table 3:** Details of grazing system, concentrate feeding and mineral supplements

	Respondents % (n)
**Grazing system**:	
Set stocking	19 (19)
Rotational	78 (80)
Both	3 (3)
	
Mixed grazing	45 (46)
Separate grazing	52 (54)
Both	3 (3)
	
**Concentrate feeding to lambs**:	69 (70)
Start pre-weaning	71 (47)
Fed to appetite	44 (30)
<20 kg	38 (26)
>20 kg	19 (13)
	
**Administered minerals**:	81 (84)
Cobalt	70 (73)
Copper	29 (30)
Selenium	26 (27)
Other	16 (17)
	
**Method of administration**:	
In feed	19 (17)
Separate dose	57 (50)
With anthelmintic	7 (6)
Some combination of 2 of the above choices	17 (15)

Details on additional nutritional supplementation (concentrate/minerals) practices are shown in Table 3. The majority, 69% (70) of respondents reported feeding concentrates to lambs at grass (Table 3), which began preweaning in 71% of cases (Table 3). Sheep were supplemented with extra minerals on 81% (84) of farms. Cobalt was the predominant mineral given (70% [73]) followed by copper (29% [30]), and selenium (26% [27]). A smaller number of respondents (16% [17]) indicated giving minerals other than those specified (Cobalt, Copper or Selenium).

### Control of gastrointestinal parasites

**Figure 1:**
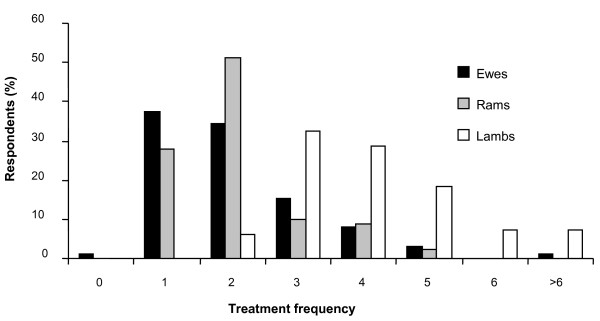
**Annual frequency of anthelmintic treatments administered to ewes, rams and lambs**.

Details of treatment practices are shown in Table 4. The vast majority of farmers indicated that they followed a set treatment programme for their stock. The estimated number of treatments given to ewes, lambs and rams in a 'normal' year are shown in Figure 1. Both ewes and rams were treated less frequently than lambs with the majority of ewes and rams receiving one or two treatments. A substantial proportion, 61% (60), administered 4 or more treatments to lambs in a 'normal' year. The most popular time indicated by the respondents to treat ewes was at housing (68% [71]), and premating (55% [57]) (Table 4). The majority (72% [75]) indicated dosing lambs before moving to aftergrass (Table 4). The majority of respondents indicated that they would treat all ewes (97% [98]) or lambs (100% [101]) in a particular group at any given time as opposed to selectively treating some individuals.

**Table 4:** Treatment practices for gastrointestinal nematodes for the different age and sex of the sheep

	**Lambs %** **(n)**	**Ewes %** **(n)**	**Rams %** **(n)**
	
**Dose according to**:			
Set programme	86 (87)	94 (88)	93 (83)
Sign of disease	14 (14)	6 (6)	7 (6)
			
**Time when animals dosed**:			
At housing	15 (16)	68 (71)	44 (46)
Before moving to aftergrass	72 (75)	20 (21)	23 (24)
Pre-mating		55 (57)	60 (62)
Pre-lambing		14 (14)	
Post-lambing		32 (33)	
			
**Weight basis for dosing**:			
Heaviest actual	54 (55)	33 (32)	ND*
Heaviest guessed	29 (30)	43 (42)	ND
Average guessed	16 (16)	22 (22)	ND
Heaviest: actual + guessed	1 (1)	1 (1)	ND
Combination of all 3		1 (1)	ND
			
**Withold food prior to dosing**:	30 (31)	27 (28)	ND
>12h	7 (2)	12 (3)	ND
Keep in after dosing:	27 (28)		ND

* ND = no data.

A summary of responses in relation to how the amount of anthelmintic administered to ewes and lambs was calculated is shown on Table 4. Over 45% (46) of farmers (greater in ewes than lambs) indicated some element of guesswork in their weight calculations. Most respondents indicated they would weigh lambs and dose according to the heaviest lamb in the group (54% [55]) while for ewes a greater proportion of respondents indicated they would guess the weight of the heaviest ewe and dose accordingly (43% [42]). The accuracy of the dosing gun was always checked on 59% (60) of farms with 34% (35) stating they 'sometimes' checked the accuracy of the dosing gun.

The practice of withholding food before dosing lambs or ewes was reported by approximately 30% of respondents (Table 4), of whom a minority indicated the fasting period was greater than 12 hours. Twenty-seven percent (28) of respondents reported keeping their animals off pasture after treating with an anthelmintic. The duration of the holding period varied between one and six hours.

**Figure 2:**
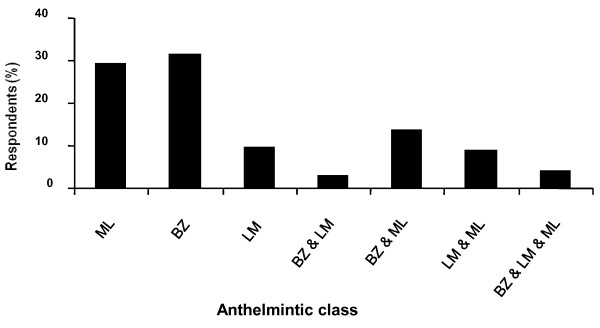
**Class of anthelmintic used in previous year**. (ML = Macrocyclic Lactones, BZ = Benzimidazoles, LM = Levamisole)

**Figure 3:**
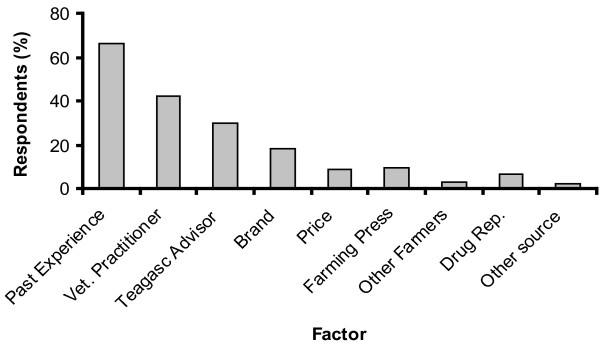
**Factors governing anthelmintic choice: the proportion of respondents who selected each factors as a 1st or 2nd choice**.

Benzimidazoles and macrocyclic lactones were the anthelmintic classes of choice on all farms, with levamisole proving the least popular of the three classes (Figure 2). The factors that respondents indicated influenced their choice of anthelmintic product are summarised in Figure 3. The majority of respondents indicated that past experience of the product was the most influential factor.

**Figure 4:**
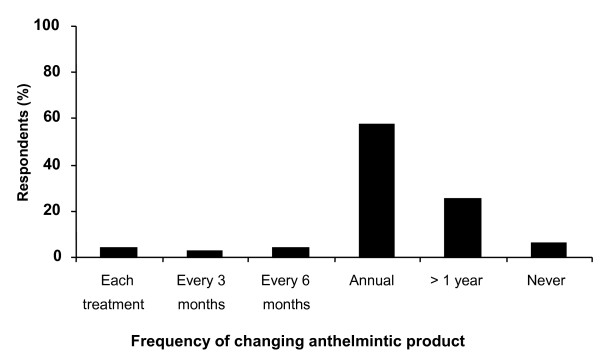
**Frequency of anthelmintic product change**.

The frequency with which farmers changed the anthelmintic product used is shown in Figure 4. The majority of sheep producers, 58% (57), indicated they switched anthelmintic class on an annual basis while 25% (25) indicated that the duration between switching was longer than one year.

Over one third of respondents (37% [38]) believed that anthelmintic products were not working as well as in previous years. Of these the majority, 82% (31), indicated that the benzimidazoles were not as effective, while dissatisfaction with levamisole and macrocyclic lactones was 32% (12) and 21% (8), respectively. Twelve per cent of respondents reported that parasite resistance to anthelmintics had been confirmed in their flock.

**Figure 5:**
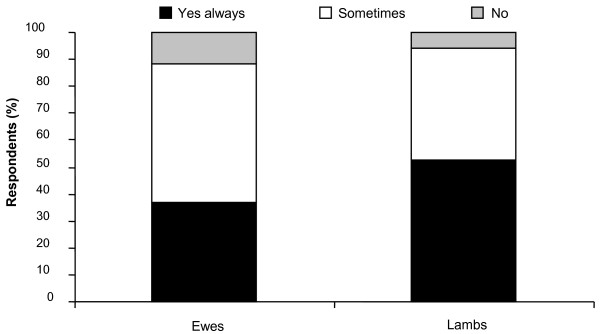
**Movement to 'clean' grazing post treatment**.

Figure 5 provides a summary of reponses regarding ewe and lamb movement to graze 'clean' pasture post- anthelmintic treatment.

The majority of respondents (94% [98]) treated purchased animals with an anthelmintic before mixing them with their own flock. Of these, 32% (29) indicated that the anthelmintic they would use would be a different product to that used for the flock in the current year. The majority of respondents (71% [20]) treated with a macrocyclic lactone.

## Discussion

From this survey it is evident that anthelmintics are an integral part of parasite control strategies. However, the gastrointestinal nematode control practices revealed by the survey highlight the need for communication on 'best' dosing practices. Departures from these 'best' practices will encourage the development and spread of anthelmintic resistance. Despite the widely available information on appropriate dosing practices the results indicate a lack of implementation of some of the basics, such as how the dose rate is determined and whether dosing equipment is checked before use. If a sub-optimal amount is administered this will increase the selection pressure for resistant worms [11-15] and result in a poor anthelmintic response, which may in turn select for anthelmintic resistance [3] and lead to the need for more dosing.

In the current climate of emerging anthelmintic resistance it is clear that parasite control practices that were used in the past and yielded favourable production outcomes in terms of managing parasites must be re-evaluated. Strategies based on suppressive and frequent treatments will select strongly for resistance and do not represent a sustainable approach to parasite control in general. Results on frequency of treatment revealed, unsurprisingly, that ewes and rams were treated less frequently than lambs for gastrointestinal parasites. The treatment frequency in lambs does suggest that a suppressive anthelmintic treatment strategy was being used on the majority of farms. Moreover, the routine treatment of adult sheep needs to be questioned. For instance, ewes pre-mating will generally have a low parasite burden and will not benefit from anthelmintic treatment. As pasture contamination in the autumn/winter will also be lower, treatment of ewes at this time will select for anthelmintic resistance as any worms that survive treatment will have a prolonged reproductive advantage and so become the dominant contributor to infection on pasture [16]. It is now regarded that treatment of ewes pre-mating should be restricted to ewes in poor condition or ewe lambs.

Withholding feed from livestock for 12 hours prior to the administration of an oral benzimidazole or macrocyclic lactone has been reported as a good practice in achieving improved efficacy of these drugs [17,18]. While 30% of respondents indicated they withheld feed prior to administration of drugs (question did not ask to specify class of drug), the withholding period was not the recommended 12 hours for the majority of respondents. Withholding food for less than 12 hours has minimal impact on anthelmintic efficacy.

Another factor considered to increase the risk of developing resistance on a farm is the inadvertent importation of drug-resistant worms in purchased sheep. The practices of treating and quarantining purchased animals and delaying the move of treated stock to 'clean' pasture are now considered important in curbing the spread of resistance [16]. While almost all farmers (94%) reported that purchased animals were treated with an anthelmintic prior to mixing with the rest of the flock, 68% indicated this would be with the anthelmintic being used in the current year. Up until recently, in light of the high prevalence of benzimidazole resistance, it was recommended that purchased sheep be treated sequentially with macrocyclic lactone and levamisole to minimise the risks involved [16]. With increasing reports of resistance to ivermectin and levamisole and the advent of a fourth class of anthelmintic on the market the advice has become more specific in that moxidectin (3-ML) and monepantel (4-AD) are ideally used as quarantine treatments. While there is no critical evidence to support the proposition that the annual alternation of anthelmintic class slows the development of resistance [11,19], results from this survey clearly indicate this message has been widely accepted.

In the past 'drench/treat and move' to 'safe' pasture was a globally recommended parasite control practice for lambs [3,20] which, providing drug efficacy was high, ensured that 'safe' pasture maintained this minimally contaminated status for a longer period thus negating the need for frequent treatment of young livestock. Van Wyk [3] has questioned the wisdom of this system, indicating that it can hasten the development of resistance as the anthelmintic resistant worms, that have survived treatment, will be the dominant contributor to the population on the 'safe' pasture. Conder and Campbell [11] state that drench-and-move systems should be considered only on a case-by-case basis and not for widespread use. Today it is commonly listed as a practice to be avoided especially on farms where resistance is a problem [3,16,21,22].

Research results have indicated that improved nutrition (metabolisable protein) enhances the host's resilience to parasites [23]. As an extension to this, one might expect that lambs being fed concentrate are less exposed to parasite challenge and as such should need fewer treatments. In this study, the absence of any evidence for this is probably a reflection of the set approach to treatment used by the majority of respondents. Moreover, it was interesting to note that while the enterprises did differ in the number of livestock units per hectare this did not impact on the number of anthelmintics administered.

Overall, the evidence indicates a need for a greater awareness of the principles that underpin the sustainable use of anthelmintics and the practices that preserve anthelmintic efficacy should be given a very high priority in the design of helminth control programmes on each farm. To this end, the potential of veterinary practitioners and agricultural advisors as sources of information on best practice should be targeted.

## Competing interests

The authors declare that they have no competing interests.

## Authors' contributions

TP Compiled the questionnaire, entered and evaluated the data and was joint lead author of the manuscript. BG, GM and TDW conceived the study, participated in the design and co-ordination. BG performed the statistical analysis and was joint lead author of the manuscript. JPH provided advice on study design, statistical analysis and participated in writing of the manuscript. TDW provided advice on data analysis and participated in writing of the manuscript. All authors read and approved the final manuscript.
